# Simulating contact networks for livestock disease epidemiology: a systematic review

**DOI:** 10.1098/rsif.2022.0890

**Published:** 2023-05-17

**Authors:** William T. M. Leung, James W. Rudge, Guillaume Fournié

**Affiliations:** ^1^ Communicable Diseases Policy Research Group, Department of Global Health and Development, London School of Hygiene and Tropical Medicine, London WC1E 7HT, UK; ^2^ Veterinary Epidemiology, Economics and Public Health Group, Pathobiology and Population Sciences Department, Royal Veterinary College, London AL9 7TA, UK; ^3^ Faculty of Public Health, Mahidol University, Bangkok 10400, Thailand; ^4^ INRAE, VetAgro Sup, UMR EPIA, Université de Lyon, Marcy l'Etoile 69280, France; ^5^ INRAE, VetAgro Sup, UMR EPIA, Université Clermont Auvergne, Saint Genes Champanelle 63122, France

**Keywords:** livestock production, network model, epidemiology, network simulation model, livestock trade, infectious disease

## Abstract

Contact structure among livestock populations influences the transmission of infectious agents among them. Models simulating realistic contact networks therefore have important applications for generating insights relevant to livestock diseases. This systematic review identifies and compares such models, their applications, data sources and how their validity was assessed. From 52 publications, 37 models were identified comprising seven model frameworks. These included mathematical models (*n* = 8; including generalized random graphs, scale-free, Watts–Strogatz and spatial models), agent-based models (*n* = 8), radiation models (*n* = 1) (collectively, considered ‘mechanistic’), gravity models (*n* = 4), exponential random graph models (*n* = 9), other forms of statistical model (*n* = 6) (statistical) and random forests (*n* = 1) (machine learning). Overall, nearly half of the models were used as inputs for network-based epidemiological models. In all models, edges represented livestock movements, sometimes alongside other forms of contact. Statistical models were often applied to infer factors associated with network formation (*n* = 12). Mechanistic models were commonly applied to assess the interaction between network structure and disease dissemination (*n* = 6). Mechanistic, statistical and machine learning models were all applied to generate networks given limited data (*n* = 13). There was considerable variation in the approaches used for model validation. Finally, we discuss the relative strengths and weaknesses of model frameworks in different use cases.

## 1. Introduction

Livestock holdings may be epidemiologically connected through both direct and indirect contacts. Direct contact typically pertains to the movement of livestock between holdings, while mechanisms for indirect contact include the transfer of biological material, equipment or personnel [1]. These contact patterns can be conceptualized as networks in which nodes may represent livestock populations (given that livestock are often managed in groups or are otherwise spatially clustered) and edges represent the contact(s) of interest between those populations. It is well recognized that the structure of livestock contact networks has important implications for infectious disease transmission dynamics [2–6]. Characterizing the structure of these networks therefore plays a crucial role in understanding transmission patterns of infectious diseases in livestock and, consequently, for informing disease risk assessments and control strategies. This may involve the use of disease transmission models which explicitly account for contact network structure [3,7–11].

Insights about the epidemiological importance of livestock contact networks, especially livestock movement (e.g. trade) networks [1,12–15], have been generated by the analysis of routinely recorded livestock movement data collected via livestock identification and traceability systems (LITS) [14,16–19]. Where such routine data are unavailable (or insufficient), targeted network surveys can also be conducted [7,20–24].

Such empirical approaches are, however, associated with major challenges. In certain settings, LITS may not be implemented as data collection, and sharing may be restricted by commercial interests and related data privacy concerns [3,25–27]. The costs and infrastructure required to implement and sustain routine systems also constrains their feasibility, especially in low- and middle-income countries [28]. The analysis and utility of such data may be constrained by its vastness [28]. Moreover, a lack of updated or complete data may also limit its use for supporting decision making during disease outbreaks [28–30]. While network surveys have been used when such data are unavailable, these are usually targeted towards specific geographical locations and time periods. Indeed, both routine and non-routine network data capture activities are highly resource intensive and are therefore likely to be targeted towards livestock species or production types of particular interest from a national livestock disease-management perspective [28,31–33].

Model-based approaches are increasingly being used to help address some of these challenges. We therefore conducted a systematic review to provide an overview of the state-of-the-art in modelling livestock contact networks. Our objectives were to identify the main types of models and methods used, compare their applications and data requirements, and examine the extent to which such models have been validated. Based on the findings, we also discuss key challenges and opportunities for future research in this area. In this review, we focus on studies which have employed empirically informed, model-based approaches of network (re)-construction or inference, with a primary interest in epidemiologically relevant (i.e. potentially infectious) contacts between livestock populations.

## 2. Methods

### 2.1. Systematic search strategy

This systematic review followed the PRISMA 2020 guidelines for the reporting of systematic reviews [34]. Search terms were developed around four key topics: (i) livestock and poultry, (ii) networks, (iii) models, and (iv) disease. Four databases—Medline, Embase, Web of Science and Scopus—were queried using title, abstract and keyword searches on 22 January 2021 and no date limits. Database searches were repeated on 27 January 2023 to cover all records published up to this date. Relevant subject headings were applied to databases using subject heading indexing (i.e. Medline and Embase; electronic supplementary material, table S1). Search terms within the ‘networks’ topic were informed by previous reviews of the use of network simulation models in different contexts [35–39]. However, broad terms were also included to ensure identified records were not restricted to known model types. Within each search topic, Boolean ‘OR’ operators were used to combine search terms and subject headings, while different topics were combined using ‘AND’ operators (electronic supplementary material, table S1). Wildcards, truncations and adjacency searches were applied using the relevant syntax for each database. Peer-reviewed papers and conference proceedings were all eligible for inclusion. The screening process was expanded to include the reference lists of the included publications, as well as any papers that cited them. For full search terms see electronic supplementary material, table S1.

### 2.2. Inclusion and exclusion criteria

Inclusion and exclusion criteria were agreed by all authors. A single reviewer screened records but discussed any records for which inclusion was uncertain with the other authors. Screening was split into two stages:

Stage 1: Titles, abstracts and keywords were screened; records were rejected if any of the following statements were true: (i) there was no reference to livestock; (ii) there was no reference to contacts between livestock, contact networks or infectious disease dynamics on networks; (iii) the record was not peer-reviewed, and (iv) the record was not written in English.
Stage 2: Full texts were screened; records were retained if all following statements were true: (i) a model was used to simulate a network of epidemiologically relevant contacts between livestock subpopulations; (ii) the model attempted to reproduce structural properties of an empirical network and/or its underlying generating mechanisms, and (iii) these properties or mechanisms were informed empirically.

Hence, we did not review records which simulated theoretical networks (e.g. to be used as reference or null models) and/or which randomized some aspects of a network to make comparisons with empirical networks (e.g. [40,41]). We also excluded studies that solely reconstructed transmission networks, since these are subsets of the contact networks which are the focus of this review. Where multiple models were used in papers, each model was screened individually for inclusion.

### 2.3. Data extraction

Information from each study was systematically recorded in a data extraction table. This was designed to record information about: (i) the type of model used; (ii) the applications of models; (iii) characteristics of the empirical network under study (livestock type, geographical location and disease focus); (iv) definition of network nodes and edges; (v) data types and variables used for model fitting, and (vi) how the performance of models was assessed (table 2). Descriptive analyses and visualizations of the frequency of key study characteristics were conducted using R v. 4.2.0 [42].

### 2.4. Model classifications

Following exploratory scoping of the literature, particularly previous reviews on network simulation models in other disciplines [36–39], we classified models into three groups: mechanistic, statistical and machine learning. Though these categories are not mutually exclusive (e.g. mechanistic model parameters may be estimated using statistical methods), they are useful for describing the general characteristics of the reviewed models, as described below.

Mechanistic models are here defined as mathematical equations *or* an algorithmic set of rules, a ‘mechanism’, used to generate a set of edges between nodes, i.e. a network. We include in this grouping mechanistic models that span from (i) abstracted and intentionally simplified ‘mathematical models’ [38], such as scale-free and small-world models (and which include the ‘probabilistic’ and ‘idealized’ models/networks described by others) [37,39], to (ii) complex agent-based models (ABMs) explicitly modelling individual-level contact processes. Notably, across both of these subgroups, the generating mechanisms may simply serve as an arbitrary algorithmic tool used to generate networks exhibiting a certain topology, or else they may be configured to reproduce the emergent processes (assumed or otherwise) that generated the observed network, that is, based on ‘first principles’ [43,44].

Statistical models describe a network as a function of factors hypothesized to be associated with edge formation. They start with observations of an empirical network and fit the parameters of a selected model framework to the data through formal statistical inference [36,38,39]. Within this group, we include standard statistical models (e.g. generalized linear models) which may be used to estimate the probability or strength of an edge between nodes given a set of covariates, in addition to network-specific statistical models which explicitly account for the dependencies inherent to network data [36,38,39].

Machine learning models learn patterns in the data without the model being specified by the user and commonly place an emphasis on predictive accuracy rather than causal inference [45,46]. These can be broadly categorized according to whether the model fitting is ‘supervised’, where the value of the dependent variable is known (i.e. data are ‘labelled’ in machine learning-terminology), or ‘unsupervised’, which use ‘unlabelled’ data and commonly include clustering algorithms [45]. In the context of network simulation, they may be used to solve classification and regression problems.

## 3. Results

### 3.1. Screening process

**Figure 1.**
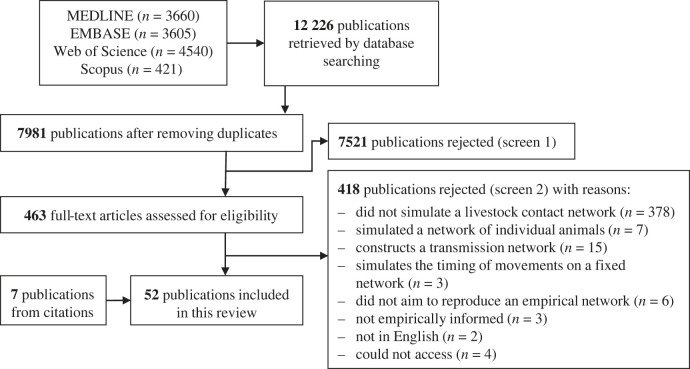
PRISMA flow diagram.

Database searches retrieved 12 226 publications of which 7981 (65%) were unique. Title, abstract and keyword screening excluded 7521 (94%) unique records (figure 1). A further 418 (5%) were excluded after screening full texts, mostly because they did not simulate a livestock contact network but presented descriptive analyses of empirical networks or simulated infectious disease transmission on empirical networks (figure 1). Six additional publications were identified from the citations of included papers. A single additional publication citing these publications was then identified. Therefore, a total of 52 publications published between 2009 and 2022 were eligible for inclusion (see electronic supplementary material, table S2 for all exclusion reasons).

To identify the number of different models used across all included studies, we considered a model to be ‘distinct’ from others when a specific framework was applied to a particular dataset. Hence, analyses in 20 publications were based on previously published models (table 2), while two publications presented multiple models, applying different model types to a single setting [47], or the same model type to different settings [29]. Consequently, 37 distinct models (tables 1 and 2) were identified and reviewed across the 52 included publications. We refer to unique models using the first published instance.

**Table 1.** Model frameworks applied to simulate livestock contact networks across 52 included studies. ABM = agent-based model; (T)ERGM = (temporal) exponential random graph model; GM = gravity model; RF = random forests.

category	model framework	number of models	number of publications
mechanistic	mathematical models	8	7
	ABMs	8	15
	radiation models	1	1
statistical	(T)ERGMs	9	7
	GMs	4	4
	other statistical models	6	17
machine learning	RF	1	1
total	-	37	52

**Table 2.** Summary of key characteristics and applications of 37 identified models. ABM = agent-based model; GM = gravity model; RF = random forests; (T)ERGM = (temporal) exponential random graph model; LITS = livestock identification and traceability system; limited data = simulating a network from the available data, when empirical networks are incompletely characterized; network-generating processes = inference of factors associated with network (or edge) generation; structure and transmission = analytical exploration of the relationship between network structure and diffusion of phenomena (e.g. disease) on networks; SA disease control = scenario analysis related to assessing the impact of disease control strategies; SA altering network = comparing the impact of alternative network configuration scenarios on simulated disease transmission patterns; SA surveillance = exploring disease surveillance scenarios; behavioural response = modelling adaptive behaviour, e.g. farmers’ response to disease on a network.

model ID		model classification			model purpose		network characteristics						data
no.	model [model name]; other papers using the model	year	model category	model framework	model applications	infectious disease model	livestock focus	setting	disease focus	nodes	edges	static / dynamic	data used for calibration
1	(Ferdousi *et al*. [48])	2019	mechanistic	generalized random graph	structure and transmission, limited data, SA: disease control	yes	pigs	USA	African swine fever	livestock holdings, markets	livestock movement	static	network survey
2	(Gates & Woolhouse [44])	2015	mechanistic	generalized random graph	SA: altering network	yes	cattle	Britain	hypothetical	livestock holdings	livestock movement	static	LITS
3	(Thakur *et al*. [47]) (A)	2015	mechanistic	Watts–Strogatz	structure and transmission, limited data	yes	pigs	Canada	porcine reproductive and respiratory syndrome	livestock holdings	livestock movement, vehicle	static	network survey
4	(Thakur *et al*. [47]) (B)	2015	mechanistic	scale-free	structure and transmission, limited data	yes	pigs	Canada	porcine reproductive and respiratory syndrome	livestock holdings	livestock movement, vehicle	static	network survey
5	(Tago *et al*. [49])	2016	mechanistic	scale-free	SA: disease control, behavioural response	yes	cattle	France	hypothetical	livestock holdings, markets, exchangers	livestock movement	static	LITS
6	(Lennartsson *et al*. [50]; [SpecNet])	2012	mechanistic	other mathematical	presents model	no	pigs	Sweden	non-specific	livestock holdings, slaughter point	livestock movement	static	LITS
7	(Rossi *et al*. [51])	2017	mechanistic	spatial	structure and transmission, limited data	yes	cattle	Italy	hypothetical highly contagious	livestock holdings	livestock movement, personnel	static	LITS
8	(Hu *et al*. [52])	2021	mechanistic	spatial	limited data	yes	pigs	China	African swine fever	livestock holdings, slaughter point	livestock movement	static	LITS
9	(Wiltshire [53]; [RUSHPNBM]) (Bucini *et al*. [54]; Koliba *et al*. [55]; Wiltshire *et al*. [27])	2018	mechanistic	ABM	SA: altering network, structure and transmission, behavioural response	yes	pigs	USA	porcine epidemic diarrhoea	livestock holdings, slaughter points, feed mills	livestock movement, feed, vehicle	dynamic	emergent
10	(Yang *et al*. [56]) (Yang *et al*. [57,58])	2019	mechanistic	ABM	SA: disease control, limited data, behavioural response	yes	cattle	USA	foot and mouth disease	livestock holdings, exchangers, markets	livestock movement, vehicle	dynamic	emergent
11	(Ross *et al*. [59])	2011	mechanistic	ABM	presents model	no	cattle	USA	bovine tuberculosis	livestock holdings, markets	livestock movement	dynamic	emergent
12	(Liu *et al*. [60]; [Epirur_Cattle])	2012	mechanistic	ABM	limited data	yes	cattle	USA	hypothetical direct contact	livestock holdings	livestock movement	dynamic	emergent
13	(Ansari *et al*. [61])	2021	mechanistic	ABM	presents model	no	pigs	Germany	non-specific	livestock holdings, exchangers	livestock movement	dynamic	LITS
14	(Brock *et al*. [62])	2021	mechanistic	ABM	SA: disease control	yes	cattle	Ireland	bovine herpesvirus type 1	livestock holdings	livestock movement	dynamic	emergent
15	(Knight *et al*. [63,64])	2021	mechanistic	ABM	structure and transmission, behavioural response	yes	cattle	Scotland	hypothetical slowly spreading	livestock holdings	livestock movement	dynamic	LITS
16	(Kim *et al*. [65]; Pomeroy *et al*. [66])	2016	mechanistic	ABM	structure and transmission	yes	cattle	Cameroon	foot and mouth disease	geo-locations	livestock movement	dynamic	network survey
17	(Kong *et al*. [67])	2022	mechanistic	radiation model	limited data	no	poultry	China	non-specific	geo-locations	livestock movement	static	emergent
18	(Valdes-Donoso *et al*. [68])	2017	machine learning	RF	limited data, network-generating processes	no	pigs	USA	porcine reproductive and respiratory syndrome	livestock holdings, markets	livestock movement	static	network survey
19	(Nicolas *et al*. [69])	2018	statistical	GM	network-generating processes, limited data	no	cattle, sheep/goats, camels	Mauritania	non-specific	geo-locations	livestock movement	static	network survey
20	(Chaters *et al*. [28])	2019	statistical	GM	limited data, network-generating processes	yes	cattle	Tanzania	non-specific	geo-locations	livestock movement	static	movement permits
21	(Qiqi Yang *et al*. [15]	2020	statistical	GM	limited data	no	poultry	China	avian influenza	geo-locations	livestock movement	static	network survey, emergent
22	(Blair and Lowe [70])	2022	statistical	GM	SA: disease control	no	pigs	USA	non-specific	geo-locations, slaughter point	livestock movement	static	network survey
23	(Ortiz-Pelaez *et al*. [71])	2012	statistical	ERGM	network-generating processes		sheep/goats	Ethiopia	non-specific	geo-locations	livestock movement	static	network survey
24	(Relun *et al*. [29]) (A)	2017	statistical	ERGM	network-generating processes	no	pigs	Bulgaria	non-specific	livestock holdings, exchangers	livestock movement	static	LITS
25	(Relun *et al*. [29]) (B)	2017	statistical	ERGM	network-generating processes	no	pigs	Spain	non-specific	livestock holdings, exchangers	livestock movement	static	LITS
26	(Relun *et al*. [29]) (C)	2017	statistical	ERGM	network-generating processes	no	pigs	France	non-specific	livestock holdings, exchangers	livestock movement	static	LITS
27	(Kukielka *et al*. [32])	2017	statistical	ERGM	network-generating processes	no	pigs	Georgia	African swine fever	geo-locations	livestock movement	static	network survey
28	(Poolkhet *et al*. [72])	2018	statistical	ERGM	network-generating processes	no	poultry	Thailand	avian influenza	livestock holdings, exchangers, markets, slaughter point, other	livestock movement, other	static	network survey
29	(Belkhiria *et al*. [73])	2019	statistical	ERGM	network-generating processes	no	cattle, sheep/goats, donkeys	Senegal	Rift valley fever	geo-locations	livestock movement	static	network survey
30	(Hammami *et al*. [74])	2022	statistical	ERGM	network-generating processes	no	pigs	France	non-specific	livestock holdings, slaughter point	livestock movement	static	LITS
31	(Lee *et al*. [75])	2021	statistical	TERGM	structure and transmission, SA: disease control	yes	pigs	Vietnam	African swine fever	livestock holdings	livestock movement, indirect	dynamic	network survey
32	(Lindström *et al*. [25]; [USAMM]) (Brommesson *et al*. [76]; Buhnerkempe *et al*. [77,78]; Gilbertson *et al*. [79]; Gorsich *et al*. [80,81]; Kao *et al*. [82]; Sellman *et al*. [83]; Tsao *et al*. [84])	2013	statistical	statistical other	SA: disease control, limited data, SA: surveillance	yes	cattle	USA	non-specific, foot and mouth disease, bovine tuberculosis	geo-locations	livestock movement	static	movement permits, census
33	(Sellman *et al*. [85])	2022	statistical	statistical other	limited data	no	pigs	USA	porcine epidemic diarrhea	livestock holdings	livestock movement	static	movement permits, census
34	(Lindström *et al*. [86]) (Brommesson *et al*. [87]; (Lindström *et al*. [88–90])	2009	statistical	statistical other	network-generating processes, structure and transmission	yes	cattle, pigs	Sweden	non-specific, hypothetical	livestock holdings	livestock movement	static	LITS
35	(Xiao *et al*. [91]) (Pomeroy *et al*. [66])	2015	statistical	statistical other	network-generating processes	yes	cattle	Cameroon	foot and mouth disease	geo-locations	livestock movement	dynamic	network survey
36	(Moon *et al*. [26])	2019	statistical	statistical other	limited data	no	pigs	USA	non-specific	livestock holdings	livestock movement	static	census
37	(Schumm *et al*. [92])	2015	statistical	statistical other	limited data	no	cattle	USA	non-specific	geo-locations	livestock movement	static	census

Following the PRISMA checklist, we highlight nine studies that might appear to meet the inclusion criteria, but were excluded. Three studies rewired empirically observed networks without also attempting to simulate the empirical network [93–95]. Three studies simulated the timing or volume of livestock movements on a predefined (non-modelled) network [96–98]. Two used mechanistic models with entirely hypothetical parameter values [99,100]. One study applied random forests (RFs) to predict the timings of trading events, without using this information to simulate a network [101].

### 3.2. General model characteristics

**Figure 2.**
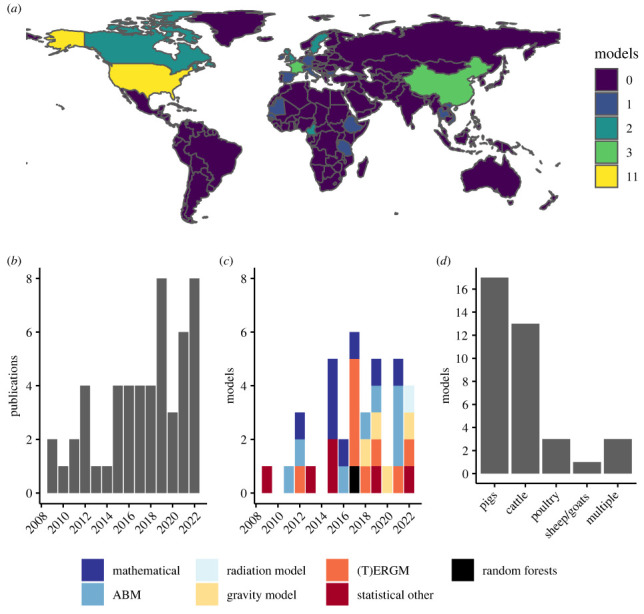
Scope of included papers and models: (*a*) map of countries models were applied to; (*b*) papers published by year; (*c*) models published by year according to model group (blue = mechanistic, red/orange = statistical, black = machine learning); (*d*) livestock types models were applied to.

The identified models were applied to 20 countries in four continents; no eligible models were applied to Australia or South America. The USA was the most well-represented country, with 11 distinct models applied (figure 2*a*). Most models were applied to a single livestock type, including pigs (*n* = 17), cattle (*n* = 13) and poultry (*n* = 3). Three models were applied to multiple livestock types (figure 2*d*). All models were applied in a disease context, related to specific (*n* = 18), non-specific (*n* = 13) or hypothetical diseases with specific characteristics (*n* = 6). Infectious disease transmission was simulated on the networks generated by 18 models (13 mechanistic; 5 statistical; table 2).

In 25 models, nodes represented farms or herds, with 14 of these also accounting for other units such as markets, slaughterhouses and/or livestock traders. Nodes were livestock populations in given administrative areas (e.g. villages, provinces and counties) in the other 12 models. Edges represented livestock movements in all models: either movements of animals among populations (*n* = 24), or transhumant movements of whole livestock populations between geographical areas (*n* = 3). Seven models simulated multi-layer networks with additional sets of edges representing epidemiologically relevant contacts via vehicles, personnel or feed providers. A single model broadly defined an edge as any type of potentially infectious contact in the context of avian influenza without defining transmission routes specifically [72]. Most models (*n* = 27) generated static networks. However, the timing of trades on the simulated static network was sometimes time varying, e.g. based on a probability of trading [49]. Alternatively, nodes or edges were sometimes added or removed by copying empirical records exactly (i.e. without modelling these) [44,52]. Contrastingly, eight ABMs and two statistical models generated dynamically evolving networks.

Most models were statistical (*n* = 19), with the most common frameworks being exponential random graph models (ERGMs; *n* = 9), gravity models (GMs) (*n* = 4) and other statistical models (*n* = 6). Only one machine learning model, based on RFs, was identified. The mechanistic models (*n* = 17) included mathematical models (*n* = 8), ABMs (*n* = 8) and a radiation model (*n* = 1) (table 1). The first model was published in 2009, but most (*n* = 31; 84%) were published between 2015 and 2022 (figure 2*c*).

In the following sections, we first review the objectives addressed by the different model frameworks and the data sources used. We then introduce the key methodological characteristics of each modelling framework, including how they have been calibrated to data, and review the degree to which their performance was assessed.

### 3.3. Model applications

**Figure 3.**
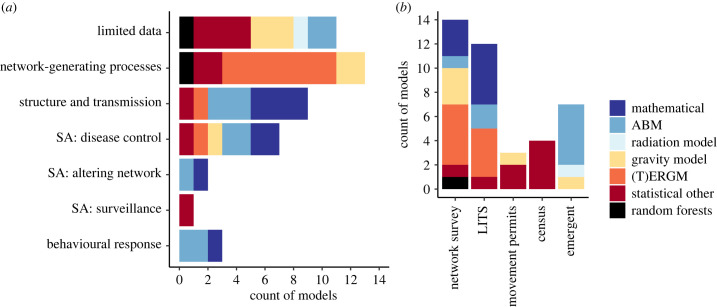
Model applications and data sources by model framework. (*a*) Model applications (multiple permitted): limited data = simulating a network from the available data, when empirical networks are incompletely characterized; network-generating processes = inference of factors associated with network (or edge) generation; structure and transmission = analytical exploration of the relationship between network structure and diffusion of phenomena (e.g. disease) on networks; SA disease control = scenario analysis related to assessing the impact of disease control strategies; SA altering network = comparing the impact of alternative network configuration scenarios on simulated disease transmission patterns; SA surveillance = exploring disease surveillance scenarios; behavioural response = modelling adaptive behaviour e.g. farmers' response to disease on a network. (*b*) Data sources used for model calibration. Blue = mechanistic; red/orange = statistical; black = machine learning. LITS = livestock identification and traceability systems; emergent = did not use network data but instead used data to parametrize model processes influencing edge formation.

Network simulation models were used for a range of applications which varied according to the model type used (figure 3*a;* table 2). For 13 models, multiple applications were identified.

Approximately half of models (16/37) were used to generate networks based on limited data, for example where total network data was not available but descriptive statistics of that network were, or where models based on complete networks were used for prediction in other settings. These included all model frameworks described above, except ERGMs. A single study used artificially constrained data on indirect contacts among farms to explore how inferring these contacts using different levels of information and assumptions influenced the outputs of disease transmission models [51].

A third of models (*n* = 13), mostly statistical (*n* = 12), were applied to explore network-generating processes, specifically, the inference of factors associated with network (or edge) generation. For the RF model, the relative importance of predictors was assessed by comparing prediction accuracies of models with and without a given predictor. Nine models, mostly mechanistic (*n* = 7), were applied to analytically explore the relationship between network structure and diffusion of phenomena (e.g. disease) on networks.

Models were also applied to test scenarios related to (i) assessing the impact of disease control strategies (*n* = 7) such as targeted livestock movement restrictions, culling or vaccination; (ii) using simulated livestock movement patterns to inform optimal sites for directing disease surveillance activities (*n* = 1; [80]), and (iii) comparing the impact of alternative network configuration scenarios on simulated disease transmission patterns (*n* = 2; all mechanistic). These scenarios involved, for example, rewiring nodes [44] and changing the composition of the farm population [53]. Mechanistic models were applied to explore the interaction between agents' adaptive behaviour, and network formation or disease spread. Examples of such applications included modelling of farmers’ decisions to implement biosecurity measures in response to disease risk [54], or trigger sales in anticipation of movement restrictions [49]. In Knight *et al*. [64], farmers' adaptive behaviour (i.e. anticipatory response to disease control interventions) influenced the formation of the network itself.

Three models were presented as a proof of principle to demonstrate their ability to reproduce structural features of an empirical livestock contact network, without further application [50,59,61]; these models were therefore omitted from figure 3*a*.

### 3.4. Data sources used

Different data sources were exploited for calibrating models, with some variation seen between model types (figure 3*b*). Most models (*n* = 30) were informed by empirical network data, including data from network surveys (*n* = 13), LITS (*n* = 12), censuses with some data on livestock trade, i.e. capturing total number of animals ‘sold or moved’ by actors in a given year (*n* = 2), and livestock movement permits which are used in some countries for recording and regulating movements, e.g. across administrative borders (*n* = 1; figure 3*b*). Contrastingly, mechanistic models sometimes did not use network data, but instead used data to parametrize model processes influencing edge formation (e.g. herd demographic processes (*n* = 6; table 2)).

While models sometimes exploited similar data types, the way that these data were used to calibrate models varied substantially according to model type as detailed in the next section (§3.5).

### 3.5. Model frameworks

#### 3.5.1. Mechanistic

##### 3.5.1.1. Mathematical models (*n* = 8)

###### 3.5.1.1.1. Generalized random graphs

Random graphs generate edges between sets of nodes at random, either by assigning a fixed number of edges [102,103] or by assigning edges with a fixed uniform probability [104]. These models therefore control for network density alone, and the resulting networks fail to capture some important structural features of empirical networks, especially high clustering and a right-skewed degree distribution [38,105,106].

Generalizations may, however, be applied to control for other network structural features beyond density thus permitting the generation of more ‘realistic’ networks [38,106]. The configuration model, or matching algorithm [107,108], allows for degree distribution to be fixed by algorithmically assigning a number of incoming and outgoing connections (stubs) to nodes, while randomly matching in- and out-stubs between different nodes. Other structural features can be controlled for: for example, in the pig movement network generated in Ferdousi *et al*. [48], connections were only permitted between certain stub combinations, thus additionally controlling for selective mixing among nodes (assortativity). Gates & Woolhouse [44] also adopted a modified configuration algorithm to generate cattle trade networks, preserving farms' empirical daily amounts of purchases and sales, while selectively matching those reported to have exchanged cattle of the same type (dairy/beef) in the same market, on the same day.

###### 3.5.1.1.2. Scale-free models

Other types of mathematical model seek to reproduce stylized topologies that are common in empirical networks. A key example is the scale-free property which results from the network degree distribution following a power law: *p*_k_ ∼ *k*^−γ^; where *k* denotes degree and *γ* the scaling parameter. The Barabasi & Albert [109] preferential-attachment model generates scale-free networks by progressively adding nodes to a network, with new nodes preferentially forming edges with high-degree nodes. This generates hub-like structures observed in many empirical networks, including those of livestock, where most nodes are poorly connected and a small number of nodes (e.g. markets and breeding farms) have a very high number of connections [105,110].

Thakur *et al*. [47] used the Barabasi–Albert model to simulate scale-free pig trade networks, fitting the model with a scaling parameter derived from empirical studies. The resulting network was altered in a second step by randomly rewiring edges connecting node types that were not connected in the empirical network, while preserving clustering coefficient and mean degree of the Barabasi–Albert model simulation. Tago *et al*. [49] generated a scale-free cattle trade network using an empirically derived scaling parameter and mimicked the real network by classifying nodes as markets, dealers or farms, based on the degree of these nodes determined empirically.

###### 3.5.1.1.3. Watts–Strogatz model

The Watts–Strogatz model is another example of a model which reproduces particular features of empirical networks—in this case, ‘small-world’ properties. The latter refers to networks with short average path lengths, as observed in random graphs, but with higher clustering than is found in random graphs of equivalent size and the same mean degree [111].

This is achieved by taking a ring lattice network, which exhibits high clustering, and randomly rewiring a proportion of its edges such that average path length is reduced. The edge rewiring probability (*p*) is the single parameter by which the network can be interpolated between the highly clustered lattice and random graph [36,105]. Thakur *et al*. [47] used this model to generate pig trade networks, choosing a value for *p* to reproduce clustering coefficients observed in empirical networks.

###### 3.5.1.1.4. Other mathematical models

Other network simulation model frameworks have been devised within different fields of study. Lennartsson *et al*. [50] describe an algorithm which generates spatially explicit networks of a defined number of nodes and mean degree which can then be tuned to target specified levels of degree-assortativity (selective mixing between nodes of similar degree), clustering coefficient, fragmentation index and spatial aggregation of nodes (random to aggregated). As a proof of principle, the authors generated networks matching values of these statistics as observed in an empirical swine transportation network.

###### 3.5.1.1.5. Spatial models

With the models described above, the influence of nodes' spatial locations is irrelevant for edge formation. In reality, however, the probability of a connection between livestock populations is likely to be influenced by the geographical distance between them [51,86,112]. While distance may be a variable in other types of model, some of the simplest spatial models express the probability of an edge between nodes as a function of distance alone. For example, in Hu *et al*. [52], edges between nodes were simply assigned if the Euclidean distance was lower than an empirically informed threshold. In Rossi *et al*. [51], the probability of contacts between farms via veterinary staff visits was estimated by fitting a logistic regression with distance as the predictor variable.

##### 3.5.1.2. Agent-based models (*n* = 8)

In ABMs, a set of autonomous agents interact with one another and their environment according to defined rules and processes [113,114]. A key feature of ABMs is that they allow complex phenomena to emerge from such processes [114]. Indeed, a livestock contact network can be considered to emerge from the multitude of economic, demographic, husbandry or other behavioural processes occurring at the level of individual agents operating in the system. This may be explicitly modelled within an ABM framework.

In six identified ABMs, network evolution was driven by herd demographic processes (e.g. livestock births, ageing/growth and deaths), and agent trade or partnership generation processes (e.g. selection of trade partners according to geographical distance, and compatibility in terms of industry role and current need to buy or sell) [53,56,57,59–62]. In these models, agents could be defined with distinct industry roles, holding capacities and geographical locations. In an additional model layer in Liu *et al*. [60], individual animal contacts during grazing were modelled using random walks. In Knight *et al*. [63,64], a dynamic trade network was generated from defined partnership rules—with the rate at which trade partnerships formed and dissolved, dependent on farms’ in- and out-flow of animals (i.e. supply and demand). In the most recent paper, farm-level demand, and consequently farmers' edge forming and dissolving behaviours, were adaptive to market shocks such that farms with high-demand sought partnerships at a higher rate. Kim *et al*. [65] simulated a population of mobile pastoralist agents based on seasonal movement rules informed by field surveys. Edges (contact between herds via grazing) were then considered between agents setting up camp within a given distance from one another.

##### 3.5.1.3. Radiation models (*n* = 1)

Radiation models, which were initially developed in the human mobility literature as an alternative to GMs ([115]; see next section), represent a mechanistic approach to predict human movements based on population distributions alone (i.e. distance is not used directly). This method takes analogy from radiation emission and absorption processes in physical sciences and was initially used to describe human commuting patterns, with commuters being ‘emitted’ from an origin and ‘absorbed’ by employment opportunities [115]. The model stipulates that the commuting flow (*T_ij_*) between an origin (*i*) and destination (*j*) is a function of the size of their respective populations (*m_i_* and *n_j_*) and, notably, the ‘intervening opportunities' between *i* and *j* (alternative employment sinks). The latter are represented by the population (*s_ij_*) in the area of the circle with radius *r_ij_*, centred at *i* (excluding *m_i_* and *n_j_*) (equation (3.1)). The variable, *T_i_* represents the overall count of individuals starting their journey at location *i* $\left(T_{i}\equiv \sum_{\;j\neq i}T_{ij}\right)$, which is taken as a proportion of *m_i_*.

3.1 $$\left\langle T_{ij}\right\rangle =\;T_{i\;\;}\frac{m_{i}n_{j}}{\left(m_{i}+s_{ij})(m_{i}+n_{j}+s_{ij}\right)}.$$

Kong *et al*. [67] adapted the radiation model to predict country-scale poultry flows in China, with poultry population representing supply (*m_i_*), and human populations representing demand (*n_j_*) and ‘intervening’ demand (*s_ij_*).

#### 3.5.2. Statistical

##### 3.5.2.1. Gravity models (*n* = 4)

GMs were initially developed to model the flow of commodities between pairs of discrete geographical areas (*C_ij_*, from origin *i* to destination *j*) as a function of their distance (*d_ij_*) and Gross National Products representing supply (push) at origin and demand (pull) at destination (*p*_i_ and *p*_j_), with normalizing constant *k* and coefficients *α*, *β* and *γ* (equation (3.2)) [116,117]. The standard formulation of the flow of commodities from node *i* to *j* (*C_ij_*) is

3.2 $$C_{ij}=k\frac{\;p_{i}^{\alpha }p_{j}^{\beta }}{d_{ij}^{\gamma }}.$$

This concept has been applied to model livestock trade as a function of livestock population at an origin (supply) and human population at a destination (demand) [15,28,69,70], with different functional relationships (e.g. exponential and power law) between distance and flows having been investigated [15]. Beyond the basic principles of mass and distance, the actual specification of GMs has been loosely defined [118]. GMs may be parametrized in equation (3.2) by fixing the coefficients *α*, *β* and *γ*; an approach which essentially represents a mechanistic parametrization. More often, however, these coefficients are estimated by statistical inference. For example, Qiqi Yang *et al*. [58] used both mechanistic and statistical GM parametrizations to model poultry movements. Equation (3.2) is commonly linearized by logarithmic transformation allowing additional covariates, hypothesized to be relevant for edge formation, to be included in the model (equation (3.3)).

3.3 $$\log(C_{ij})=k+\alpha \log(\;p_{i})+\;\beta \log(\;p_{j})+\gamma \log(d_{ij})+\ldots \;$$

The coefficients of such models may then be estimated by ordinary least-squares (OLS) regression (e.g. [28,69,70]).

##### 3.5.2.2. Exponential random graph models (*n* = 9)

Under an ERGM formulation, the observed network is considered as just one realization of possible networks (configurations of edges given a set of nodes) with certain characteristics that result from an unknown stochastic process [119]. The ERGM defines a model of this network generation process and a probability distribution over all possible networks. Parameters are selected and estimated, such that the probability of the observed network being generated under the defined model is maximized. It may take a general form as in equation (3.4). Here, the dependent variable is the *whole network* (the probability of drawing the observed network *y* from the distribution *Y*), which is modelled as a function of covariates *z_k_(y)* hypothesized to be relevant for network formation. The covariates are weighted by coefficients *θk*, with *c* being a normalizing constant [119,120].

3.4 $$P_{\theta }(Y=y|n\;\mathrm{nodes})=ce^{\theta_{1}z_{1}(y)+\ldots +\theta_{k}z_{k}(y)}.$$

A model with a covariate for network density alone is equivalent to a random graph model [119]. However, additional covariates may describe attributes of edges, nodes or notably, local structural features, such as the tendency for reciprocated edges, or the tendency for triangles to form (i.e. where three nodes are completely connected) [121]. Network simulation is achieved by drawing from the probability distribution of possible network configurations given a set of nodes and their attributes. This is the basis for model fitting and assessment of goodness-of-fit: coefficients are fit and the model goodness-of-fit checked based on comparison between characteristics of the simulated and empirical networks [122]. ERGM output is analogous to a logistic regression making their interpretation straightforward [29,71].

ERGMs have been fitted to networks of livestock movements between aggregated spatial units [32,71,73], or actors such as livestock holdings [29,72,74]. These models have sometimes been applied to livestock networks of entire countries (e.g. [29,74]). The use of ERGMs in this context has allowed livestock contact networks to be modelled and simulated as a function of the tendency of farms to form (dis-)assortative trade partnerships with respect to farm size, type, management practices, company affiliation or location [29,74], in addition to local structural factors [29,32,71,73].

An extension of ERGMs, temporal exponential-family random graph models (TERGMs), enables the statistical modelling of tie dynamics [123]. Here, ERGMs are used to model both tie formation and dissolution, with potentially distinct models for each process. Separable-TERGMs (STERGMs) are used in the latter case. While these models were developed for the statistical modelling of empirical dynamic networks, model parameters may alternatively be defined without being inferred statistically i.e. similar to mechanistic modelling. Lee *et al*. [75] applied TERGMs in this way to simulate dynamic contact networks among pig farms according to a defined mean degree (overall and by node type) and the frequency of contacts.

##### 3.5.2.3. Other statistical models (*n* = 6)

In a series of developments, [86–90] applied a hierarchical Bayesian model to Swedish pig and cattle movement networks incorporating data on between-holding distances, origin and destination production types, and the number of animals in each holding.

Building on these, the USAMM model [25], which has been applied and modified extensively [76–84], uses a Bayesian kernel approach to reconstruct the US cattle trade network. Similarly to GMs, movement probabilities were modelled as a function of the number of cattle premises at the origin and destination, and the distance between them, while also incorporating data on historical state-level cattle inflows. Sellman *et al*. [85] adapted these methods to reconstruct the national US pig movement network.

Xiao *et al*. [91] modelled pastoralists' movements by fitting statistical models to detailed movement survey data. Distinct seasonal movement trajectories were modelled according to different movement models. For example, origin-destination movements were modelled using a Brownian bridge motion model. This movement model was used to generate dynamic daily contact networks among mobile herds in a separate study [66], with ‘contacts’ between herds being considered when pastoralists set up camp within a given distance from one another on a given day—corresponding to grazing distances observed in field surveys.

Moon *et al*. [26] and Schumm *et al*. [92] used a statistical inferential method of maximum entropy (which is designed to estimate probability distributions from highly dimensional data) to estimate the movement probabilities of pigs within and between geographical units from census data. Based on the size and number of farms within each county, these movement probabilities were then used to simulate a farm-to-farm pig movement network.

#### 3.5.3. Machine learning

##### 3.5.3.1. Random forest (*n* = 1)

The probability or strength of an edge between two nodes can be treated, respectively, as a classification or regression problem which may be addressed using machine learning models such as classification or regression tree-based approaches. These models perform repeated partitions of the data based on the values of predictor variables, such that the observations in each partition are increasingly homogeneous with respect to the outcome of interest [124]. The values of observations in the resulting terminal tree-nodes are used as the basis of prediction. RF models combine multiple trees to reduce the variance of predictions and increase predictive performance [124,125]. Predictors may take the form of node or edge attributes. Valdes-Donoso *et al*. [68] used a RF to classify whether livestock movement occurred between pairs of nodes (farms or markets) as a function of geographical distance, node type mixing patterns (i.e. farm, market) and whether or not nodes were under shared ownership. This fitted model was then used to predict edges among nodes in the larger region, for which relevant node attributes were available.

### 3.6. Model validation

Adopting definitions by Porgo *et al.* [126], model validation (i.e. ‘how well a model performs and how applicable the results are to a particular situation’) was performed for around two-thirds (23/37) of models. We do not consider model calibration here (see §3.5). There was considerable variation in the methods by which model performance was assessed. This extended from the types of network properties considered, the methods of validation used, and the rigour to which this was carried out.

In terms of the types of validation used, 17 models were internally validated, while nine were externally validated. Approaches for external validation included splitting the data into training and validation sets (e.g. [68]), or through comparison with different datasets [15,56,65,67], such as for different time points [74,85,87]. A single GM was externally validated by assessing whether observed changes in livestock movements resulting from demand changes (i.e. closure of a terminal swine-processing facility) could be reproduced in the model [70]. Lastly, for two models, cross-validation was performed by comparing networks simulated by different models [65,91].

Regarding the types of network statistic considered, a third of models were validated by comparing structural network statistics of simulated and empirical networks (*n* = 14; electronic supplementary material, table S3). For example, model goodness-of-fit for ERGMs (*n* = 9) was assessed by comparing distributions of structural metrics not used for calibration such as in- and out-degree, geodesic distances, edgewise shared partnership and triad census.

Other models were internally validated at the level of the dyad (*n* = 6). Examples of approaches here included computing the predictive accuracy of binary or weighted edges based on, respectively, the area under the receiver operating characteristic curve, or correlation coefficients ([69] GM, [25] other statistical, [68] RF; Kong *et al*. [67] radiation model). Distributions of observed and predicted geographical distances between connected dyads were also sometimes compared ([86] other statistical, [68] RF, [56] ABM).

Alternatively, the outcomes of epidemics modelled on simulated networks were compared (*n* = 4) with either (i) epidemics modelled on empirical networks ([51] spatial), or (ii) empirical disease incidence. For example, the outputs of epidemics simulated on the pastoralist ABM by Kim *et al*. [65] were compared with annual disease incidence data. Meanwhile, Qiqi Yang *et al*. [58] assessed the statistical association between a GM-inferred poultry trade network and the geographical distribution of different avian influenza virus subtypes.

## 4. Discussion

In this systematic review, we present an overview of empirically informed, model-based approaches of network generation and inference that have been applied to simulate networks of contacts between livestock populations. We found 52 publications presenting 37 distinct models and seven model frameworks being used in this context. The increasing number of publications identified over the past decade illustrates the growing interest in this area. This reflects the considerable interest in applying network science to study the contact networks of livestock more broadly [2,3].

All models were applied to generate insights relevant to livestock diseases, with nearly half being used as inputs of infectious disease transmission models. However, the reviewed models varied greatly in their formulation, complexity and realism, use of data, and in the methods by which their performance was assessed. Consequently, we now turn to a comparison of model frameworks and discuss how their particular features can present opportunities and challenges in different use cases. Finally, we discuss issues and possible solutions around model assessment and validation.

A major application of reviewed mathematical models was to explore the relationship between network structure and disease transmission dynamics. Indeed, the relative simplicity of some of these models and, in particular, their ability to yield analytical solutions, lends them towards such applications. These types of models have consequently been applied extensively to explore the diffusion of phenomena on networks in the network literature [38,105,106]. This simplicity—in particular the ability of these models to be calibrated using few parameters—has also resulted in their application towards generating networks when empirical data are limited [47,48,51], or else totally absent, through the adoption of hypothesized parameter values (e.g. [99,100]). Mechanistic approaches, such as ABMs and radiation models, can also be used in cases where network data are unavailable but the processes underlying the formation of the network are understood and can be parametrized, i.e. based on first principles.

Notably, mechanistic models based on first principles may be more suitable for extrapolating beyond the data to which they were calibrated [127]. Hence, by altering their generative rules, such models can be applied to explore, for example, how counterfactual network configuration scenarios influence disease transmission dynamics [53]. Explicit modelling of the assumed generative mechanisms of the network further allows for an examination of its emergent properties. This makes it possible to explore realistic farm (or node) level disease control interventions that act to modify network structure [44]. Importantly, such approaches also allow complex adaptive properties of the system to be explored [113]. This includes agents' behavioural adaptations as a response to disease [49,54], or as an unintended consequence following regulatory changes or top-down interventions (e.g. [64]), as has been observed empirically [128–131].

Despite these important functions, purely mechanistic approaches commonly rely on calibration to select structural features (e.g. degree distribution and clustering coefficients) with no attempt to assess whether these features are necessary, or adequate, for representing an empirical network [36,39]. A comparative strength of statistical network models lies in their utility for assessing which features are relevant for network generation, as well as allowing for a measure of the uncertainty of these estimates given the data [36,38–40,132]. This also allows networks to be simulated while accounting for and incorporating this uncertainty which, in the context of infectious disease modelling, can help avoid overfitting epidemic outcomes to observed networks [39,133,134]. Despite this utility, less than a third of models being applied to simulate networks for infectious disease modelling were statistical models, with the remaining being mechanistic. This may broadly reflect the contrasting applications of these different model groupings in our included studies; namely, the emphasis on hypothesis testing for the statistical models, particularly ERGMs which were the most well-represented model framework in this grouping.

As noted, the major application and strength of statistical models reviewed here was the inference of factors associated with network formation. An important limitation that was not addressed in the reviewed literature is that traditional statistical methods, such as GMs using OLS specifications, assume statistical independence between observations. Due to dependencies inherent to network data, such assumptions may not hold, potentially resulting in biased estimates and hence predictions [40,116,135,136]. While standard OLS specifications of GMs cannot explicitly model these dependencies, corrections have been proposed to account for the effects of assumptions about non-independence (summarized by Broekel *et al*. [116]). However, to our knowledge, these have not been used in GMs applied to livestock contact networks.

A major strength of ERGMs lies in their ability to explicitly model and account for such dependencies; networks can be modelled and simulated as a function of parameters describing structural characteristics (e.g. transitivity or mutuality effects) in addition to node and edge factors [120,136,137]. ERGMs are therefore a powerful means of assessing the statistical significance of a range of factors on edge formation, as well as for simulating networks from these parameterizations. In practice, however, it is not always possible to generate a well-fitting model. This can be due to issues with ‘model degeneracy’ which can occur when high correlations between network effects result in unrealistically dense or sparse networks [29,120,136].

We identified a single model applying RFs to predict and simulate livestock contact networks. More broadly across the network simulation modelling literature, a variety of supervised machine learning approaches have demonstrated high predictive utility when applied to the movements of humans [138,139] and wild animals [140]. Given increasingly widespread application of machine learning approaches across the network prediction literature and the growing volume and complexity of livestock data, including movement data [141], there is likely to be considerable scope in applying machine learning methods to predict and simulate livestock contact networks.

This review has highlighted significant variation in how models were calibrated and assessed. This is of course strongly reflective of the availability of empirical network data and the purpose or intended application of models. In the context of simulating networks relevant for epidemiological study, however, given the fundamental relationship between network structure and disease transmission dynamics, it is clear that meaningful and realistic outputs rely on simulated networks accurately reproducing epidemiologically relevant features of the empirical networks. A remaining challenge then is understanding which structural features are epidemiologically relevant, and which we should therefore seek to reproduce. Indeed, the importance of these features may be highly disease and context specific [121,122,134,142]. Calibration and validation based on a few select network statistics is unlikely to be sufficient to reproduce networks exhibiting similar structure and diffusion patterns as their empirical counterparts [143–145]. Comparisons based on multiple structural characteristics are likely to be more robust, especially when the selection of these metrics is based on their relevance for diffusion processes, as is routine practice for ERGMs [121,122]. A highly valuable and interpretable form of validation, where data are available, is the comparison of epidemic outcomes on simulated and empirical networks. Comparison of simulated and observed disease incidence or prevalence is also particularly valuable, given that a transmission network is necessarily a subset of the potentially infectious contact network [146].

This review has some limitations. Despite our efforts to keep search terms broadly relevant to network simulation modelling, the lack of standardization in terminology means additional papers may have been missed using our search criteria. We have adopted the term ‘network simulation model’ from Bellerose *et al*. [35] and suggest its use in future publications on this topic. This would help to make this area of research more visible and avoid overlap with the related, yet distinct, context in which the term ‘network modelling’ is commonly applied, i.e. simulating disease spread on (empirical or simulated) networks. To keep the scope adequately focused and the synthesis feasible, we have focused on models which were used to simulate empirical-like and empirically informed contact networks of livestock populations. Hence, we highlight that this review does not present a complete compendium of all possible modelling frameworks, nor was it intended to. Alternative frameworks could be identified from the broader literature, such as from related reviews on network simulation models in other contexts [35–39].

This review serves to synthesize and categorize the heterogeneous group of models that have been applied to simulate the contact networks among livestock populations in the context of livestock disease epidemiology. Despite the important remaining challenges with model validation, this review highlights a number of unique functions afforded by network simulation models which enable us to advance beyond simple descriptive analyses of livestock networks, or infectious disease modelling on empirical networks. With increasing recognition of the need for evidence-based approaches to livestock production and health, particularly in the context of multitudinous high-profile, and often economically devastating, livestock and zoonotic disease outbreaks in recent decades, it seems reasonable to assume that efforts towards livestock network data collection will continue to gain ground. The types of modelling approaches reviewed here are well positioned to derive key insights from this data. Furthermore, such models can be used to inform the design of future empirical studies and livestock tracking systems, in order to optimize their efficiency and utility in generating data needed for effective disease surveillance and control [26,28].

## Data Availability

The data extracted for this review and the R code used to generate the figures in this review are available from https://github.com/wtm-leung/Network-modelling-review and are archived on Zenodo (https://doi.org/10.5281/zenodo.7883259). Database search terms and a full list of full-text articles assessed for eligibility are provided in the electronic supplementary material [147].
